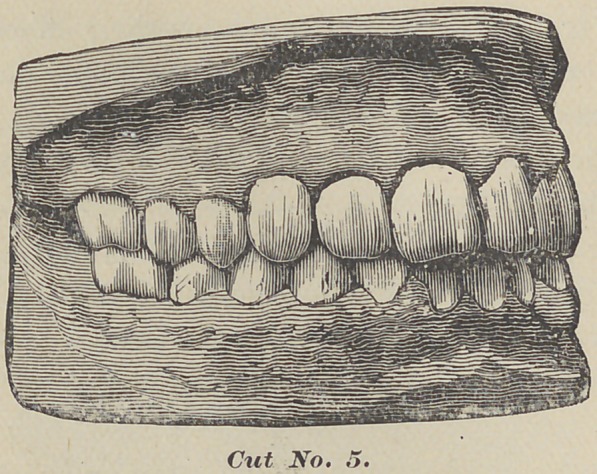# Irregularities

**Published:** 1887-04

**Authors:** G. W. Keely


### THE DENTAL REGISTER.

VOL. XLI.] APRIL, 1887. [No. 4.

### COMMUNCATONS.

### Irregularities of the Teeth.

BY G. W. KEELY, D.D.S.

Read at the 43rd Annual Meeting of the Mississippi Valley Dental Association.

GENTLEMEN:—There are a great many interesting points on this question of irregularites of the teeth to which I would like to call your attention, but on this occasion, will refer briefly to a few of them. We all recognize the fact that an "ounce of prevention is better than a pound of cure," hence the necessity of the watchful care of the intelligent operator over his little patients during the period of second dentition. The temporary teeth are as important to the child for masticating purposes, and the consequent preservation of health, as the permanent ones are to the adult. And, if possible, every one of them should be preserved until their mission is fully accomplished. Very many otherwise intelligent parents do not realize this fact, and the child is never taken to the family dentist until after some of the teeth have exposed pulps and are painful. And, as a rule, nothing will satisfy them short of the extraction of the offending member. Premature removal of these teeth often cause serious trouble.

While we claim emphatically that all should be preserved, we assert, without the fear of successful contradiction, that the premature loss of the cuspids and molars will invariably cause a crowded condition of the permanent teeth.

It is common to see the permanent incisors crowded when first erupted, and if we lose sight of the fact that the child is growing and the arch is enlarging, we may wrongfully imagine it necessary to remove the cuspids to give ample room for the incisors to fall into line. Their removal would allow them to do this, but would result in a far greater trouble in the near future. The first permanent bicuspids usually erupt two or three years before the permanent cuspids. When the temporary cuspids are prematurely extracted, the result is that the space left will cause the earlier eruption of the first bicuspid, and it will, as a rule, come in contact with the lateral incisor, thus crowding the permanent cuspid wholly outside the arch. We must keep vividly in mind the *fact* that the permanent cuspids play a conspicuous part in the contour of the face, and are considered the most important of the permanent set. When the permanent cuspids erupt partly or wholly outside the arch, and a thoughtless dentist commits the unpardonable sin of extracting them, he destroys the contour and beauty of his unfortunate patient's face during life.

Here is the diagram of the teeth of a boy twelve years of age,

*Cut No. 1.*

whose temporary cuspids were prematurely extracted to relieve a crowded condition of the permanent incisors, resulting in wholly crowding outside the arch both superior cuspids.

The arch is well developed, and all the teeth antagonized perfectly. In this case it was possible to expand both arches sufficiently to make room for the cuspids to come in place. But to do so, it would be necessary to break up the attachment of every tooth in the mouth. It must be borne in mind, that to widen both arches enough for the cusps of the superior teeth to antagonize naturally outside the cusps of the inferior, would have been a matter of doubt, and perhaps ended in failure, besides consuming much time and causing considerable pain and inconvenience to the boy. It must also be remembered that the cuspids require as much space in the arch as the central incisors, and when the cuspids are removed the contour of the face is destroyed beyond possible restoration. In this case the points of the cuspids had just appeared—(but cannot be seen in the cut). Both arches being well developed, the first bicuspids were removed, and in less than two years after the cuspids had taken the place made vacant by the bicuspids without any outside assistance, and the arch had expanded one-eighth of an inch in the region of the bicuspids and molars, as shown in

*Cut No. 2.*

Here is another case of a girl aged fifteen, to illustrate the evil consequences of the premature extraction of the temporary cuspids. It can be seen in this cut No. 3, that the laterals and first bicuspids are in contact.

*Cut No. 3.*

The temporary incisors were not removed until after some of the permanent ones made their appearance inside. After they were erupted in a crowded condition, the left lateral being caught inside the inferior teeth, her dentist becoming alarmed, extracted the temporary cuspids, causing the earlier eruption of the first permanent bicuspids, and they came forward and in contact with the lateral incisors, thus wholly crowding outside the arch both permanent cuspids. As we had but little time to treat this case, we extracted the first bicuspids, expanded the second, and at the same time started the cuspids back, working most vigorously on the left one to get room to take the lateral out of its lock. When this was accomplished, a retaining plate was inserted to hold the bicuspids and lateral in their new positions, leaving the process of nature to carry the cuspids in place. She was well developed for one of her age, and this deformity was very marked and mortifying to a handsome girl just bursting into womanhood.

We advised her to frequently press the cuspids back with the thumb and finger, to hurry them in place. I met her for the first time two years after, when her refractory teeth were in line, and she was transformed into a beautiful woman.

There seems to be few in our profession who fully realize the *great importance* of preserving temporary molars until after the first *permanent ones* are fully erupted and in contact. It is a wise provision of nature in furnishing these molars for masticating purposes before the loss of the anterior temporary teeth.

If they are removed prematurely, it will cause the earlier eruption of the *first permanent ones*, and they will come forward and take up a *part* or the whole of the space due the second bicuspids, and consequently cause a crowded condition of the anterior teeth. From an experience of over twenty years, I have never seen a a case where one or more of these molars were prematurely extracted, that failed to cause trouble to a greater or lesser extent. My rule is to treat such teeth, and if we can do no more than retain the roots, they will do good service in keeping the erupting first molar in place. The importance of preserving the temporary molars has but recently been brought to the attention of our profession, so far as I am aware.

We realize fully the patience it requires to properly treat them, and the many difficulties in the way, and at times the temptation is strong to apply the forceps, as the easiest way out, but we must, nevertheless, bear in mind the evil result sure to follow.

Here was shown the model of the teeth of a girl aged eleven, whose inferior temporary molars were prematurely extracted, causing the permanent molars to come forward, and the anterior teeth to fall back, the molars and first bicuspids being in contact, and the second bicuspids making their appearance wholly outside the arch. While in the superior arch the temporary molars were still in place, the space left between the anterior teeth made enunciation difficult, and greatly marred the expression of the face. The superior temporary molars were extracted, and soon after the second bicuspids were erupted. As the posterior molars occluded perfectly, a system of wedging was commenced between the first inferior molars and first bicuspids, to force the anterior teeth forward (as they did not occlude to prevent this), to get space to allow the imprisoned bicuspids to fall into line. This was accomplished, reducing the space between the anterior teeth, improving enunciation, and greatly beautifying the features. Another model of a superior arch. On one side the temporary molar was prematurely extracted, causing the first permanent one to come forward, and the anterior teeth on that side to fall back, the molar and first bicuspid being in contact, and the second bicuspid *wholly inside the arch.*

Here are other models showing a similar evil result. If I succeed in convincing one of you of the great importance of preserving these temporary molars until their mission is accomplished, I shall think I have done some substantial service.

Great good can be accomplished by carefully watching our little patients during the period of second dentition. The roots of pulpless teeth do not absorb, though pressed upon ever so hard by the erupting permanent one, but will turn it aside, either outside or inside the arch. The pulpless tooth is an obstruction in such cases, and its timely removal will allow the incoming one to fall into its normal position. (Here models were shown to illustrate this point.)

We often see the permanent incisors erupting inside the temporary ones by removing the latter. The incoming teeth will assume their normal position. If there seems to be danger of them being caught inside the inferior ones, instruct the patient to frequently press them out with the thumb, or give them a stick made of wedge wood to use as an incline plane. But if we find them caught to any considerable degree, they must then be gradually moved to their normal position by a mechanical appliance.

Lastly, I wish to call your attention briefly to what should be done with badly demoralized first permanent molars of our young patients. The removal of the first molars for the purpose of regulating teeth is a matter of no small importance. No teeth play a more conspicuous part in preserving the contour of the face, unless it be the cuspids. I never saw a case where the removal of the cuspids was indicated, nor do I ever expect to. Neither have I ever seen a case in which I would remove the first molars if they were all sound and in a healthy condition. But with one or more badly decayed and broken down, with exposed or dead pulps, and the patient under twelve years of age, the case is wholly different. Often the removal of a molar on one side will cause the anterior teeth to fall back and greatly mar the contour of the face on that side. Here is a diagram to illustrate: The gentleman is aged thirty. When sixteen, his right superior first molar was extracted, causing the second and third molar to come forward, and the anterior teeth to the central on this side, to fall back. The second molar and second bicuspid are squarely in contact, and there is a space between the central half the width of the inferior one. There is a depression on this side giving a disagreeable expression, while on the other side, where all the teeth are in place, it looks different, giving a natural expression. He is a public speaker, and the space between the centrals seriously interferes with correct enunciation. This would not occur, provided all the posterior teeth antagonized perfectly, holding the teeth to the front. Many of our little patients between the age of eight and ten have very defective first molars, broken down and with exposed pulps, which should be removed, even when there is no appearance of a crowded condition. When they are removed prior to the eruption of the second molar, the latter will come forward bodily, without the slightest indication of tilting, and stand squarely up to the second bicuspid, looking like a first molar. Then it will cause the earlier eruption of the third molar, and it is thus given space, and will be as good and perfectly developed as its neighbor.

Third molars have but few friends, for the reason they often cause serious trouble during eruption, or their effort to do so, and are so often defective, caused by their imprisoned condition. They are always good when they have ample space to erupt.

The following case is of a miss aged twelve. The first inferior molars were badly decayed and abscessed when she came to me. The superior ones could have been filled, but, owing to the crowded condition of the anteror teeth, we desired to remove all four. A few days after cut No. 4 was taken.

*Cut No. 4.*

Had it been possible to save the inferior molars (as also the superior), it would have been necessary to expand both arches to relieve the crowded condition of both the superior and anterior teeth. And it is no easy task to move pulpless teeth any considerable distance and hope to see them become firm, even after being held in position for years.

In this case we proposed to allow "Dame Nature" to bring all the teeth in line, but we soon found the second molars, just appearing, were coming forward so fast that that we found it necessary to wedge the bicuspids back to allow room for the four cuspids to fall into line. Nothing further was done but to insert a thin vulcanite plate, bearing hard on the superior bicuspids to expand them a trifle, to complete a more perfect antagonism.

Two years after the following was taken, showing both the superior and inferior teeth in line. The superior bicuspids are now one-eighth of an inch wider apart (measuring from right to left on palatine surface), than in the first model.

*Cut No. 5.*

My patient's pretty face has been made beautiful. This case paid me better than any one I had treated before, being fully appreciated both by the mother and daughter—there being no pecuniary consideration.

Here are three diagrams of a case somewhat similar, of a girl aged ten. The first molars were wholly demoralized, and the anterior teeth crowded, permanent laterals twisted, temporary cuspids in place, some of the bicuspids erupting. After the first model was taken, the four first molars were removed. One year after the second model was taken, showing the second molars almost in contact with the second bicuspids, the twisted laterals in line, and the bicuspids two-thirds developed. Two years later the third model was taken, showing all the teeth in contact, no appearance of the molars telling, as they stand up squarely to the second bicuspids.

All the teeth are regular. All I did in this case was to remove the first molars, leaving the rest to the process of nature. So, gentlemen, you can see how perfectly she has performed the work. This girl inherited a crowded condition of her teeth from her mother, her father having normal arches.

## Figures and Tables

**Cut No. 1. f1:**
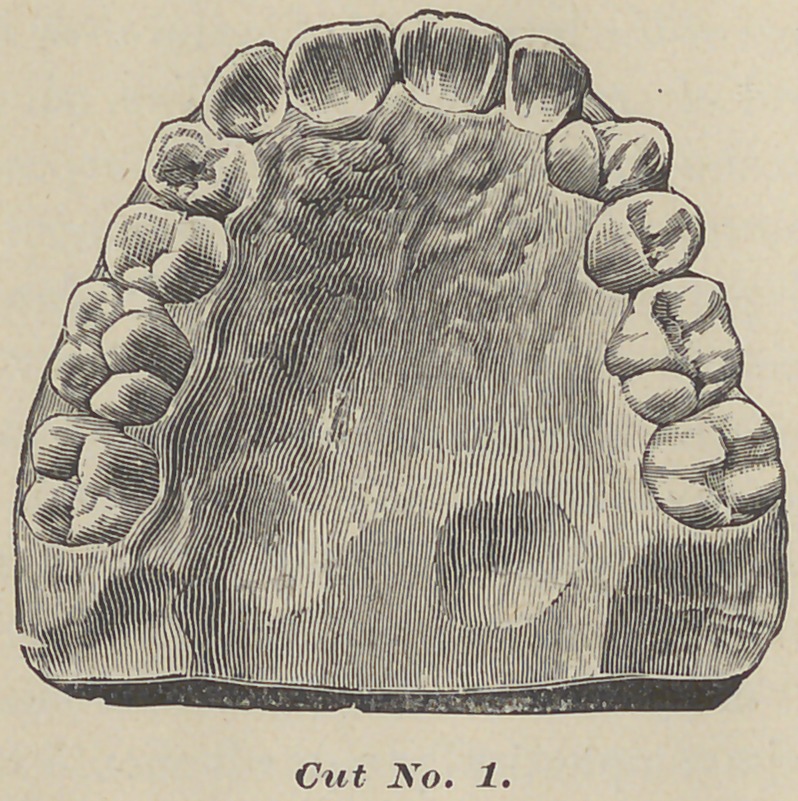


**Cut No. 2. f2:**
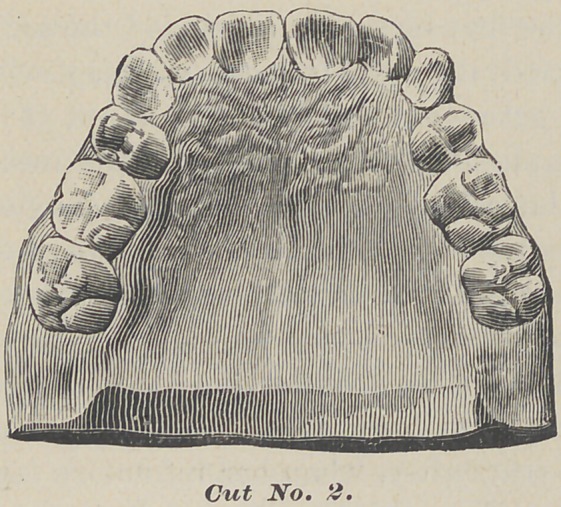


**Cut No. 3. f3:**
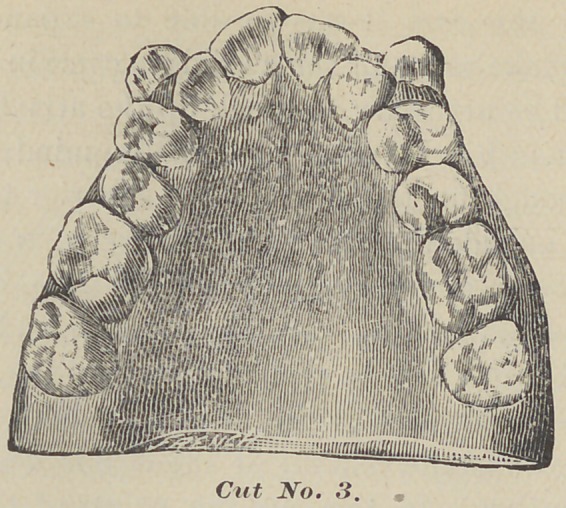


**Cut No. 4. f4:**
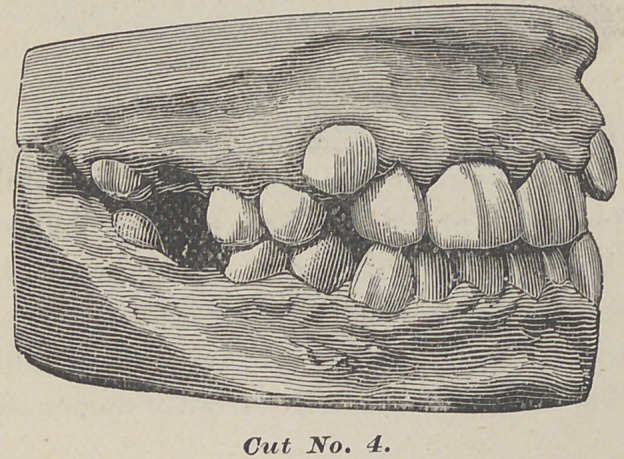


**Cut No. 5. f5:**